# Epidemiological characteristics of human psittacosis in Guangzhou, China, January 2021 to June 2024

**DOI:** 10.3389/fpubh.2025.1526990

**Published:** 2025-03-25

**Authors:** Yunjing Wen, Wei Zhang, Yongguang Li, Xinlong Liao, Jianxiong Xu, Ruonan Zhen, Pengzhe Qin

**Affiliations:** Guangzhou Center for Disease Control and Prevention, Guangzhou, China

**Keywords:** psittacosis, metagenomic next-generation sequencing (mNGS), rural areas, *Chlamydia psittaci* (*C. psittaci*), avian-to-human transmission

## Abstract

**Background:**

Psittacosis is a global and underappreciated zoonosis, with increasing reported cases in many countries. There have been several outbreaks and even deaths of psittacosis reported in China. Understanding its epidemiological characteristics and dimensions is crucial for formulating precise prevention and control strategies. This study aimed to analyze the epidemiological characteristics of human psittacosis in Guangzhou, China.

**Methods:**

The demographic characteristics, clinical manifestations, temporal patterns, geographic distribution and potential exposures of psittacosis in Guangzhou were analyzed based on the surveillance data and epidemiological investigation conducted between January 2021 and June 2024. Seasonal and trend decomposition using LOESS was applied to decompose the number of psittacosis cases into trend, seasonal and remainder component.

**Results:**

A total of 148 cases were reported, with a significant increase in the number of psittacosis cases over the study period. Most of cases were sporadic and detected by metagenomic next-generation sequencing (mNGS). Psittacosis was predominant males aged 40–79 years. Fever and pneumonia were the most commonly observed clinical manifestations. A seasonal trend was observed in the number of psittacosis cases with a high prevalence of cases in December and March. A total of 108 local cases (87%) occurred in rural regions. Among local cases, 67.7% reported a history of contact with birds or poultry, and 17.7% had been exposed to a related environment. The suspected source of infection differed between urban and rural areas, with parrots being the primary source in urban areas and poultry in rural areas.

**Conclusion:**

Increasing clinicians’ awareness, enhancing epidemiological surveillance, paying close attention to the epidemic in rural areas, and implementing measures against avian influenza, will be conducive to preventing and controlling psittacosis.

## 1 Introduction

Psittacosis, commonly referred to as parrot fever, is a zoonotic infectious disease caused by *Chlamydia psittaci* (*C. psittaci*). More than 450 bird species, including poultry, are susceptible to *C. psittaci* infection (1) and the prevalence of *C. psittaci* infections in birds is estimated to be approximately 20% (2). The pathogen can be excreted in the urine, feces and other excretions of infected birds such as chickens, ducks, pigeons and parrots, and can remain infectious in the environment for months. Humans can be infected by inhaling aerosols containing *C. psittaci*, with manifestations from mild symptoms to severe pneumonia (3). Psittacosis has been proven to be an important cause of community-acquired pneumonia (CAP), but patients are often misdiagnosed and under inappropriate treatment due to nonspecific symptoms (4). The public health risk and significance of psittacosis remain largely underappreciated.

In recent years, the development of metagenomic next-generation sequencing (mNGS) and commercial polymerase chain reaction (PCR) detection reagents has significantly increased the detection rate of *C. psittaci* (5, 6). Several outbreaks and even deaths of psittacosis reported in China, including in Zhejiang province (7), Shandong province (8) and other places, which raised public concern. However, routine surveillance of psittacosis has not been implemented in China, as it is not a notifiable infectious disease (9). Since 2021, Guangzhou, for the first time, has started to require medical institutions at all levels to implement the all-reporting mechanism for psittacosis cases to strengthen disease surveillance. Epidemiological investigations were conducted to identify suspected infection source and clusters of cases. In this study, we aim to elucidate the epidemiological characteristics of human psittacosis in Guangzhou and to provide scientific evidence for future policymaking.

## 2 Methods

### 2.1 Study areas

Guangzhou, the capital of Guangdong province, is located in the south of China, with a resident population of 18 million. The city covers an area of 7434.4 km^2^ and comprises six urban administrative regions (Yuexiu, Liwan, Tianhe, Haizhu, Baiyun, and Huangpu) and five rural administrative regions (Panyu, Huadu, Nansha, Zengcheng, and Conghua).

### 2.2 Data collection

This study was conducted by collecting data of psittacosis in Guangzhou between January 1, 2021 and June 30, 2024. Psittacosis cases data were extracted from the China Information System for Disease Control and Prevention (CISDCP). CISDCP is an Internet-based real-time system for infectious diseases in China established in 2004. Medical institutions in Guangzhou were required to report psittacosis cases to CISDCP upon obtaining a positive result via any of the following methods: detection of (1) *C. psittaci* by culture, or (2) *C. psittaci* gene fragments by mNGS in specimens, like broncho-alveolar lavage fluid (BALF), blood, or sputum, meeting the criteria for a positive mNGS result, or (3) the *C. psittaci* genome by PCR. Guangzhou Centre for Disease Control and Prevention (CDC) and the local CDC conducted epidemiological investigations on all psittacosis cases or their relatives during the study period. The information of psittacosis included demographic characteristics (e.g., age, sex, occupation, address), exposure history, symptoms, date of illness onset, date of medical institutions diagnosed and reported, types of specimens, and the mNGS examination results (if available).

### 2.3 Statistical analysis

This study described the demographic characteristics, clinical manifestations, temporal patterns and geographic distribution of psittacosis cases. Categorical variables were presented as percentages. Continuous variables were presented as means ± standard deviation (SD) for normal distribution, or as medians with inter quartile range (IQR) for non-normal distribution. Seasonal and trend decomposition using LOESS (STL) was performed to decompose the number of psittacosis cases into trend component, seasonal component and remainder component. The chi-square test was used to assess whether the suspected infection source was different in urban and rural areas. All statistical analyses were performed using Microsoft Excel 2016 and R 4.1.1.

## 3 Results

### 3.1 Demographic characteristics

Between 1 January 2021 and 30 June 2024, 148 psittacosis cases were reported in Guangzhou, including one associated death (case fatality ratio: 0.7%). Among the 148 cases, two involved cohabiting couples, and two others involved a mother and son. Except for these two clusters, the majority of the cases (144, 97.3%) were sporadic.

Psittacosis cases were predominantly males (91, 61.5%) with a sex ratio of 1.6:1. The mean age was 59 years [standard deviation (SD) = 13, range from 21 to 93]. Middle-aged and older adult aged 40–79 years represented 89.2% of cases. Household and unemployed persons, farmers, and retirees accounted for about 70% of the psittacosis cases (Table 1).

**Table 1:** Demographic characteristics of psittacosis in Guangzhou during January 2021 to June 2024.

Patients (*n* = 148)	
Characteristics	Number of cases (%)
Sex	
Male	91 (61.5%)
Female	57 (38.5%)
Age, years	
20–29	5 (3.4%)
30–39	6 (4.1%)
40–49	21 (14.2%)
50–59	44 (29.7%)
60–69	38 (25.7%)
70–79	29 (19.6%)
80–89	2 (1.4%)
≥90	3 (2.0%)
Occupation	
Household and unemployed person	38 (25.7%)
Farmers	34 (23.0%)
Retirees	32 (21.6%)
Businessman	15 (10.1%)
Worker	9 (6.1%)
Government officials	9 (6.1%)
Medical staffs	1 (0.7%)
Others	10 (6.8%)
Clinical outcome	
Discharge	147 (99.3%)
Died	1 (0.7%)
Laboratory test	
mNGS for BALF	133 (89.9%)
mNGS for blood	6 (4.1%)
mNGS for sputum	4 (2.7%)
mNGS for BALF and blood	4 (2.7%)
PCR for nasopharyngeal Swab	1 (0.7%)

Almost all psittacosis cases (147, 99.3%) were diagnosed by mNGS, with only one case was identified by PCR through active case search. Cases were detected by mNGS for 133 BALF samples, 6 blood samples, and 4 sputum samples. Four of the cases underwent both BALF and blood mNGS testing, and the results were positive.

### 3.2 Clinical manifestations

We collected the clinical manifestations data of 140 psittacosis cases through epidemiological investigation and medical records. The most common symptom was fever, which observed in almost all cases (136, 97.1%). Of these, 107 cases reported their highest body temperature as illness onset, ranging from 38°C to 42°C. Other common symptoms were cough (70.0%), expectoration (45.7%), fatigue (40.7%), chest tightness and shortness of breath (37.1%), chills (30.7%), and headache (29.7%). Other reported symptoms included myalgia, dyspnea, dizziness, sore throat, and gastrointestinal problems (nausea and vomiting, abdominal pain and diarrhea). All cases underwent X-ray or CT scan. According to examination, all patients showed unilateral pneumonia or bilateral pneumonia. The median duration from symptom onset to diagnosis was 11 days (IQR, 7 to 16). All clinical manifestations are shown in Table 2.

**Table 2:** Clinical manifestations of psittacosis cases in Guangzhou during Jan 2021 to Jun 2024.

Patients (*n* = 140)	
Symptoms and signs	Number of cases (%)
Fever	136 (97.1%)
Cough	98 (70.0%)
Expectoration	64 (45.7%)
Fatigue	57 (40.7%)
Chest tightness and shortness of breath	52 (37.1%)
Chills	43 (30.7%)
Headache	39 (27.9%)
Myalgia	28 (20.0%)
Dyspnea	20 (14.3%)
Dizziness	19 (13.6%)
Sore throat	15 (10.7%)
Nausea and vomiting	14 (10.0%)
Abdominal pain	9 (6.4%)
Diarrhea	7 (5.0%)
Pneumonia	140 (100%)

### 3.3 Temporal patterns

**Figure 1:**
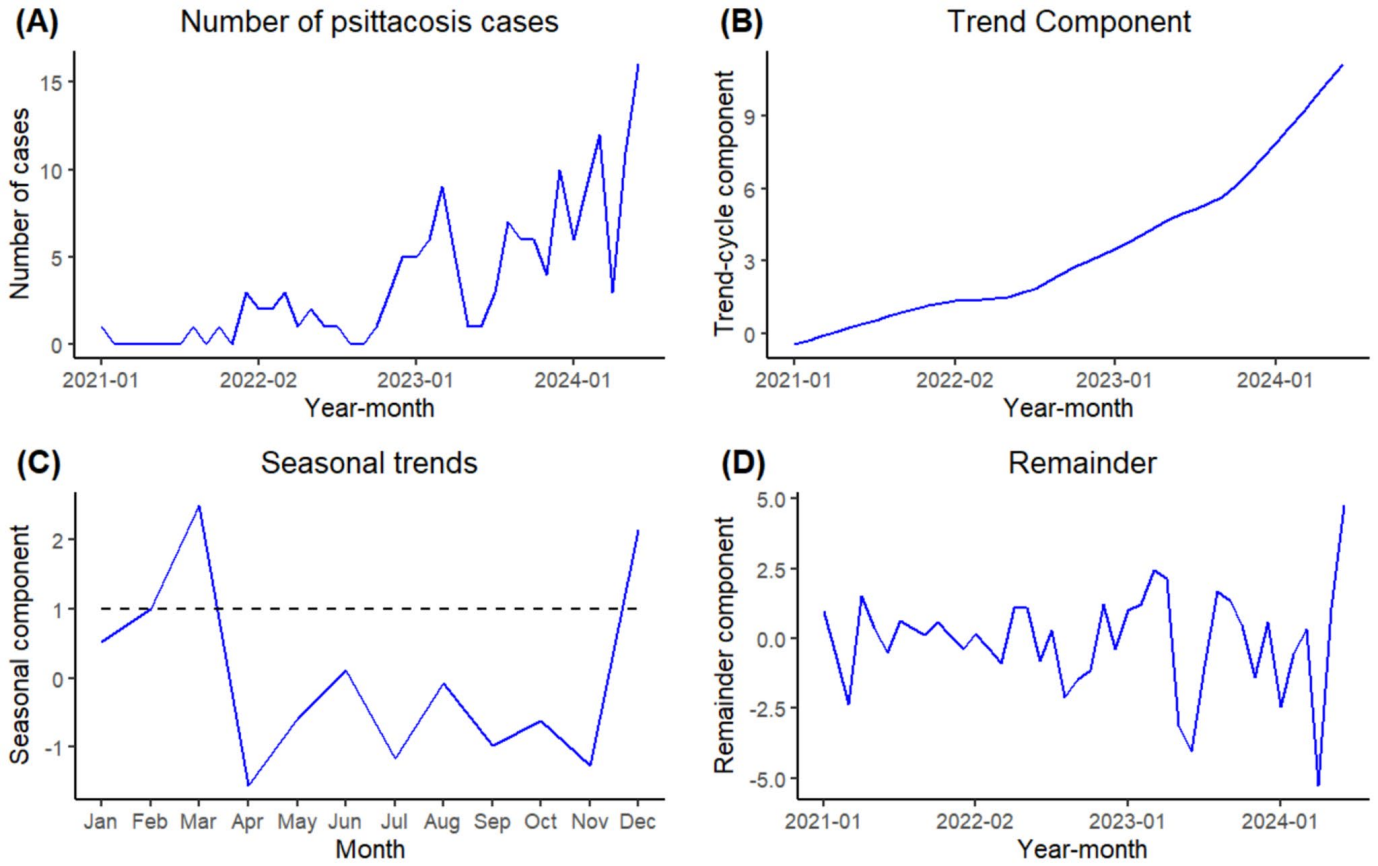
Time series of psittacosis cases number, secular trend, seasonal trend and remainder by STL decomposition. (**A**: Number of psittacosis cases; **B**: secular trend component; **C**: seasonal component; **D**: remainder component).

Figure 1A showed the change in the number of psittacosis cases in the time series between January 2021 and June 2024. The number of psittacosis cases in Guangzhou jumped from 6 in 2021 to 21 in 2022 and 63 in 2023. In the first half of 2024, 58 psittacosis cases have been reported. In Figure 1B, a significant rising secular trend was observed over the period from January 2021 to June 2024 with an accelerating growth rate. Figure 1C showed the seasonal trends of psittacosis throughout the year. The number of cases in December and from February to March were above the annual average, with *λ* values of 2.13, 1.00 and 2.48, respectively. The highest number occurred in March. Figure 1D presented the remainder component of the time series. The auto-correlation function (ACF) and partial auto-correlation function (PACF) of the remainder were performed to analyze the decomposition effect. The ACF and PACF plots showed no significant auto-correlation of the residuals. The Ljung-Box test was also applied to verify the remainder was a white noise (*p*>0.05), indicating that the decomposition effect was satisfactory.

### 3.4 Geographic distribution

**Figure 2:**
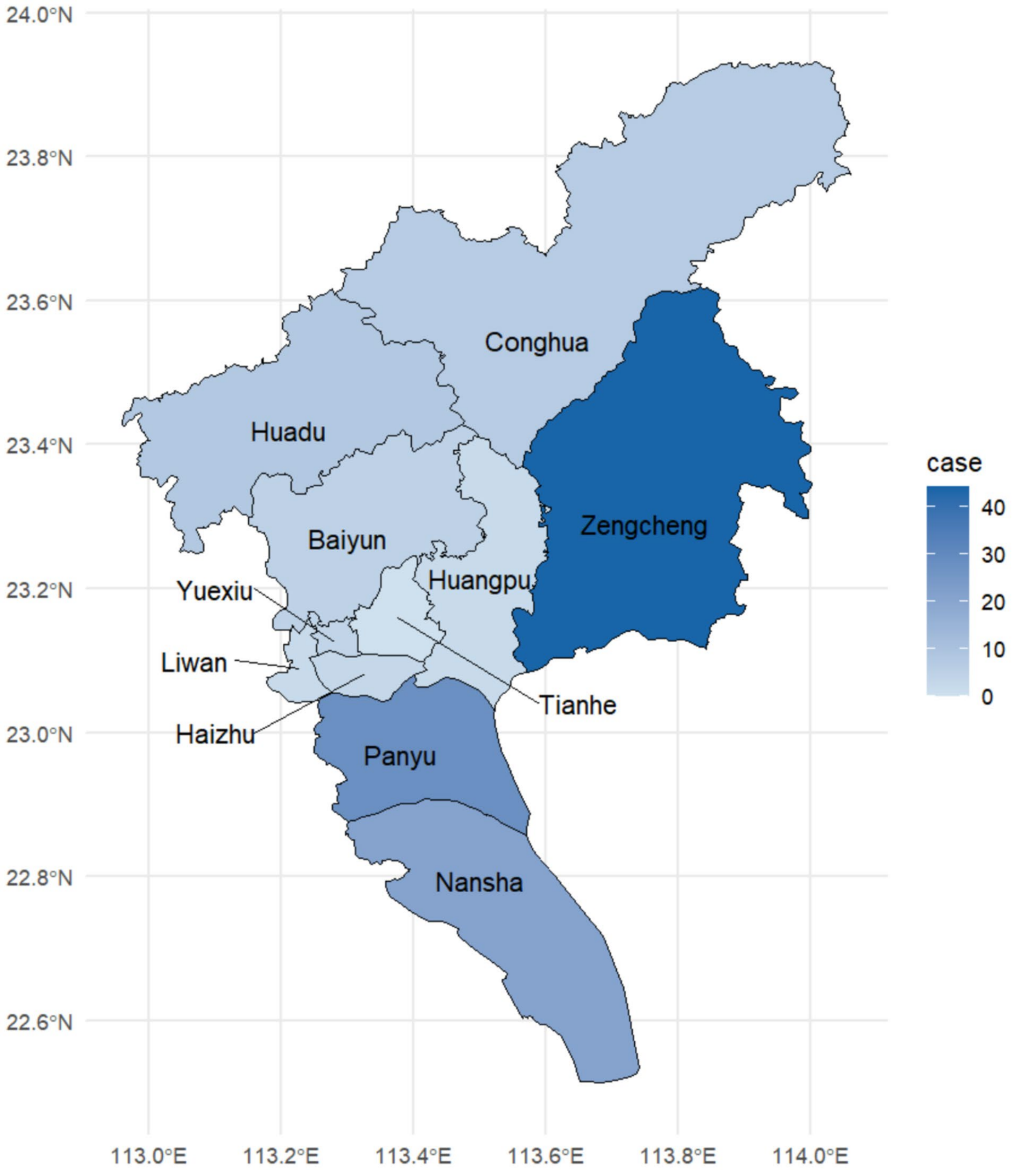
Geographical distribution of psittacosis cases by district in Guangzhou during January 2021 to June 2024.

For 148 cases, 124 were locally transmitted cases and 24 were imported from surrounding cities. During the study period, locally transmitted cases of psittacosis occurred in 10 districts in Guangzhou, with the exception of the Tianhe district. Five rural administrative regions, where had lower population density, reported significantly more psittacosis cases than the six urban administrative regions. Eighty-seven percent of cases occurred in rural administrative regions, with the highest number (44 cases) in Zengcheng, followed by 28 cases in Panyu and 21 cases in Nansha (Figure 2).

### 3.5 Suspected infection source and route of transmission

Among the locally transmitted cases, 84 cases (67.7%) reported a history of contact with birds or poultry before illness onset, and 22 cases (17.7%) did not contact birds or poultry directly but had been exposed to a related environment, such as live poultry markets, henneries, aviaries, or bird habitats. Only 16 cases (12.9%) denied having exposed to poultry or birds. The sources for the remaining 2 cases (1.6%) were unknown/unreported. Among the 84 cases with a suspected infection source, chicken (*n* = 36, 42.9%) was the most common, followed by parrots (*n* = 23, 27.4%) and ducks (*n* = 22, 26.2%). Most had contact though activities such as raising birds and poultry, slaughtering poultry, or handling infected birds. Only a small number of patients (*n* = 12, 14.3%) had one-time exposure to birds, such as buying live poultry.

The suspected infection source differed between urban and rural areas (*p* < 0.05). In urban areas, most cases (*n* = 8, 80%) reported contact with parrots with none reporting exposure to pigeons. In contrast, cases in rural areas reported more contact with poultry (*n* = 53, 71.6%) than parrots (*n* = 15, 20.3%) (Table 3).

**Table 3:** Suspected infection source of psittacosis cases in Guangzhou during January 2021 to June 2024.

Locally transmitted cases (*n* = 124)			
Suspected infection source	Urban regions	Rural regions	Overall
Contact with birds or poultry	10	74	84
Parrots	8	15	23
Pigeons	0	9	9
Chicken	2	34	36
Duck	1	21	22
Geese	0	14	14
Poultry (not specified)	1	8	9
Wild birds	1	6	7
Expose to a related environment	3	19	22
Live poultry market	2	12	14
Hennery or aviary	1	6	7
Bird habitat	0	5	5
No	3	13	16
Unknown	0	2	2

## 4 Discussion

Human psittacosis is considered an emerging public health risk. As it is not a nationally notifiable disease in China, previous studies on psittacosis were case studies (10, 11), case series analysis (12), and multi-center observational studies (13–15), which rarely described the underlying situation of the disease in China. This study is the largest cross-sectional, observational study of human psittacosis in China, which analyzed the epidemiological characteristics of psittacosis in Guangzhou from 1 January 2021 to 30 June 2024. The finding is of importance to provide information on the public health risk of human psittacosis and to formulate prevention and control strategies.

This study showed that the number of psittacosis cases in Guangzhou tripled annually since 2021, with 99.3% of cases detected by mNGS. The recent development of mNGS technology has led many hospitals to send specimens (usually BALF) from patients with unexplained severe pneumonia to third-party institutions for testing, which has directly contributed to the significant increase in the detection rate of psittacosis. There was no significant upward trend in psittacosis cases over the past 10 years in the United States and Australia (16, 17) and the annual number and notification rate in Japan declined (18). However, a similar increase in case numbers was observed in several European countries (19, 20). Austria, Denmark, Germany, Sweden, and Netherlands reported an unusual and unexpected increase in psittacosis cases from 2023 to early 2024. In Sweden, the general increase since 2017 could be attributed to the increased use of more sensitive PCR panels, whereas testing procedures of the other four European countries have not changed in recent years (20). The difference in trends between China and other countries requires further investigation. It is important to determine whether it is a true secular trend in cases or if it reflects differences in the sensitivity of surveillance systems or diagnostic techniques, as a large number of mNGS-identified cases have been reported primarily in China. The actual number of psittacosis cases in Guangzhou is likely underestimated, as most cases are reported in CISDCP after the diagnosis of pneumonia in hospitals. It is possible that cases of psittacosis remain undetected due to atypical or mild manifestations, or because they improve after anti-infective treatment without being detected, or was diagnosed as CAP. Given these factors, it is foreseeable that the number of psittacosis cases will continue to rise in the future.

Through the epidemiological investigation of psittacosis cases reported in Guangzhou, we found only two clusters. Within the two clusters, the cases both had contact with sick parrots and their illness onset occur close in time, suggesting co-exposure to infected parrots as the likely cause. Other cases did not show any family, workplace or temporal clustering, without evidence of person-to-person transmission. In addition, over 85% of locally transmitted cases reported direct contact with birds or poultry, or exposure to related environments before illness onset. This study revealed that human psittacosis is primarily an opportunistic disease caused by contacting with birds or poultry, although *C. psittaci* has the potential to evolve human-to-human transmission via various routes (8).

The epidemic process of psittacosis can be viewed as a chain with three major links. The first link is the source of infection. *C. psittaci* is a globally distributed zoonotic bacterium, and bird infections are common. While parrots have traditionally been considered the primary host of psittacosis (3), but there are also reports that poultry, turkey, pigeons, and even some mammals, such as horses (21) and sheep (22), can be infected with *C. psittaci* and be a source of infection. In Guangzhou, most psittacosis cases can be traced to poultry (especially chickens) and parrots, with a lesser number linked to wild birds and pigeons. This finding aligns with the study by Wang et al., which identified poultry as the most common source of infection in humans (51.91%), followed by wild birds (5.53%), pigeons (2.98%) and parrots (2.56%) (23). However, this result contrasts with findings from Denmark, where 80% of cases were linked to wild birds (20). This discrepancy suggested the source of infection may vary by regions, as the distribution of bird species and their populations differ significantly across areas. The source of infection was significantly different between urban and rural areas in Guangzhou. In urban areas, parrots were the most suspected source of infection, while in rural areas, poultry was more likely to be the source. The difference in the types of birds people are exposed to in urban versus rural areas may help explain this variation (24). Since much of Guangzhou’s urban area restricts live poultry operations (25), the exposure of rural people to live poultry is much more than that of urban areas.

The second link is the route of transmission. The most common route of transmission in humans is through inhalation of contaminated aerosols from feathers (e.g., flapping wings), excreta (e.g., drying), or the environment (e.g., sand and cages), as well as handling of feathers, tissues of infected birds (e.g., dissecting or eviscerating). In Guangzhou, most cases involved poultry farmers or pet parrot owners, who were likely infected through prolonged contact with birds or through close operations such as slaughtering and cleaning up feces. One case was also reported where the person was bitten by a sick parrot. Psittacosis cases who did not contact with birds or were just exposed to the related environment for a short time have also been found, but less frequently. This aligns with findings from Denmark and Netherlands, where several cases every year report no direct contact with birds (20). It suggests that the transmission of *C. psittaci* from birds (including poultry) to humans is not easy and that direct contact with infected birds or objects contaminated by their excreta remains the primary route of transmission for human psittacosis.

The third link is high-risk groups. Human psittacosis most commonly occurs in persons with a history of contact with birds or poultry, either in occupational settings or through exposure to companion bird. In Guangzhou, psittacosis cases reported were predominantly observed in males aged 40 to 79 years, and among households and unemployed persons, farmers, and retirees. This finding is consistent with the age and gender distribution observed in Japan (18), Belgium (19) and Fujian and Zhejiang, China (26, 27). We hypothesized that there were two reasons for this: first, many families in rural regions had small poultry-raising areas and middle-aged and older adults in China were more likely to be in close contact with poultry. The second was that middle-aged and older adults were more susceptible to infection because they had weaker immunity or more underlying diseases than younger people.

Human psittacosis occurred all year, with a peak season from December to March of the following year, which was consistent with other respiratory diseases. This period also coincides with the Chinese Lunar New Year, when the demand for live poultry increases, leading to more frequent transportation, trade, and consumption of live poultry. Psittacosis cases typically present with sudden onset, with fever being the most common symptom. The body temperature of fever cases was between 38 and 42°C. Another common feature of psittacosis cases was that chest radiography images show unilateral pneumonia or bilateral pneumonia. Even in the mild case found by active case search, CT scan revealed infection in the right lower lobe. A similar situation was observed in Germany, where almost all cases had pneumonia (18/19) (20). In addition to fever, some patients displayed non-specific symptoms of respiratory infection, including cough, expectoration, fatigue and chest tightness and shortness of breath. Unlike typical bacterial pneumonia, which primarily affects the lungs, systematic symptoms (like chills, headache, or muscle aches) and gastrointestinal presentations (such as nausea and vomiting, abdominal pain, or diarrhea) were also reported in this study. Clinicians should consider psittacosis when encountering patients with a history of avian or bird exposure during the peak season, especially those presenting with high fever, ineffective conventional treatments, and pneumonia seen on chest CT scans.

Geographically, the regions with the largest number of cases were Zengcheng, Panyu, and Nansha districts. Most psittacosis cases in Guangzhou occurred in rural administrative regions, where live poultry trade is permitted. This distribution is similar to that observed in Netherlands, where cases primarily occurred in poultry-dense areas, particularly in the main poultry production zones (28). It’s worth noting that this pattern was similar to the geographical distribution of human avian influenza in China in recent years (29). To prevent and control human avian influenza, almost all urban administrative regions in Guangzhou are designated as restricted zones for live poultry operations, prohibiting the trade of live poultry (25). However, Guangzhou is a big poultry-consuming city and live poultry stocks are still prevalent. Many families in rural areas operated small-scale poultry farms or keep backyard poultry (30). These families depend on poultry for income and food, even when poultry show symptoms of illness, they may still be slaughtered for sale or consumption. We analyzed the measures taken by the Guangzhou government to control avian influenza, which also helped to control the spread of psittacosis in urban areas to some extent because the two pathogens share similar routes and modes of transmission.

This study revealed the epidemiological characteristics of psittacosis in Guangzhou, China. More localized intervention policy for disease prevention and control are required. First, it is important to train medical personnel to improve the awareness and diagnostic ability of psittacosis and make full use of mNGS to screen unexplained pneumonia cases with high fever and a clear exposure history of poultry or birds. Second, both health and agriculture departments should enhance surveillance of psittacosis in humans and birds, respectively. *C. psittaci* is suggested to be included in veterinary legal quarantine and the list of notifiable infectious diseases in China. Third, the government is suggested to monitor closely the epidemic situation in rural regions and poultry-dense areas, and sustain preventive and control measures related to avian influenza (e.g., restricted zones for live poultry operations). Fourth, it should attach great importance to the publicity and health education on psittacosis among poultry farm workers, bird owners, and individuals who frequent contact with avians.

There are several limitations in this study. First, there were non-response bias and recall bias in tracing the exposure history. Second, current surveillance has found that psittacosis was mostly hospitalized and severe, but may not find some asymptomatic infection and mild cases. As a result, the true number of individuals infected with *C. psittaci* may be underestimated, and the severity of the disease could be overestimated.

## 5 Conclusion

The number of psittacosis increased rapidly in Guangzhou from 2021 to 2024, largely due to the widespread use of mNGS in unexplained pneumonia cases. The transmission route was mainly avian-to-human transmission and the peak season was from December to the following March. Most cases showed fever, pneumonia, and non-specific respiratory symptoms, with a history of contact with birds or exposure to a related environment. The majority of cases occurred in middle-aged and older adults, primarily in rural regions. In the future, increasing clinicians’ awareness about psittacosis, enhancing surveillance in humans and birds, paying close attention to the epidemic situation in rural areas, and continuing to take prevention and control measures against avian influenza, will be conducive to the prevention and control of the spread of psittacosis in humans.

## Data Availability

The raw data supporting the conclusions of this article will be made available by the authors, without undue reservation.
